# Formality in psychotherapy: How are therapists’ and clients’ use of discourse particles related to therapist empathy?

**DOI:** 10.3389/fpsyt.2022.1018170

**Published:** 2022-12-23

**Authors:** Jonathan Him Nok Lee, Harold Chui, Tan Lee, Sarah Luk, Dehua Tao, Nicolette Wing Tung Lee

**Affiliations:** ^1^Digital Signal Processing & Speech Technology Laboratory, Department of Electronic Engineering, The Chinese University of Hong Kong, Hong Kong, Hong Kong SAR, China; ^2^Department of Linguistics, University of Pennsylvania, Philadelphia, PA, United States; ^3^Department of Educational Psychology, The Chinese University of Hong Kong, Hong Kong, Hong Kong SAR, China; ^4^Department of Psychology, The Chinese University of Hong Kong, Hong Kong, Hong Kong SAR, China

**Keywords:** discourse particle, therapist empathy, formality, psychotherapy, linguistic feature, synchrony

## Abstract

**Introduction:**

Previous studies explored the preferences for therapists’ attire and office setting based on initial impressions as a reference for the formality in psychotherapy. This study examines the formality of psychotherapy by investigating therapists’ and clients’ use of discourse particles, the linguistic marker and quantifier of the formality in speech, in relation to therapist empathy in different stages of psychotherapy.

**Methods:**

Four psychotherapy sessions (representing early, mid, and late stages) each from 39 therapist-client dyads were analyzed. Trained observers rated therapist empathy in each session using the Therapist Empathy Scale.

**Results:**

Results of multilevel modeling show that synchrony in particle usage, hence synchrony in formality, between clients and therapists is not associated with therapist empathy. Therapists’ use of particles (i.e., absolute formality of therapists) was also not associated with therapist empathy. In contrast, the relative formality of therapists plays significant roles: therapist empathy is generally observed when therapists are relatively more formal than the clients (i.e., lower relative usage of particles by the therapists when compared to the clients). However, for clients who speak formally with few particles, therapist casualness (i.e., higher relative usage of particles than the clients) at the beginning of therapy may be interpreted as therapist empathy as therapists help these clients ease into the therapeutic relationships.

**Discussion:**

Our results suggest that the examination of therapists’ and clients’ use of particles across different stages of treatment may illuminate dynamic interactional styles that facilitate or hinder the psychotherapy process.

## 1 Introduction

This study explores the formality of psychotherapy by investigating therapists’ and clients’ use of discourse particles in relation to therapist empathy.

The formality of psychotherapy is an area that is not commonly explored by researchers. Previous research mainly explored therapists’ attire, which may reflect people’s preferences on the formality of therapists. Participants were asked to rate the therapists after being presented with photos of therapists wearing different attire or having a short interaction with therapists. Their results were generally equivocal: therapists with formal/professional attire were perceived as higher in expertness and credibility, whereas those with casual attire were perceived as lower in expertness (1–3). However, the participants of these studies were undergraduate students and not real clients, so their results may not be representative of clients in psychotherapy setting. Stillman and Resnick (4) also conducted a similar study in 1972 by asking 50 male students to rate 5 male trainee therapists after a 20-min initial interaction, but they found no significant effects of therapists’ attire on participants’ disclosure or perception of therapists’ attractiveness. However, as Halmagyi (5) argued, their study was conducted in early 1970s with all male therapists and participants, which may not be generalized to present times when female dominate psychotherapy and related professions. In addition, a recent qualitative study interviewed and surveyed current clients of psychotherapy and showed that clients generally perceived therapists dressed in professional/formal attire as having a more professional attitude (6).

Hubble and Gelso (7) was another study that asked about clients’ preferences in the formality of therapists’ attire. They asked 54 female undergraduate students who have real personal problems which they wanted to discuss with a therapist to engage in a 45-min interview with male therapists with three levels of attires: formal (coat and tie), casual (sport shirt and slacks), and highly casual (sweatshirt and jeans). Being undergraduate students, the clients dressed either casually or highly casually. Their results showed that clients who dressed casually preferred therapists dressing more formal than themselves with formal attire, and clients who dressed highly casually preferred therapists dressing relatively more formal than themselves with casual attire but not formal attire. In contrast to other studies that were based on forced options of *absolute formality* (i.e., binary: formal vs. casual), their findings shed light on the importance of *relative formality* (i.e., therapists being relatively more formal or more casual than their clients). Also, they were aware of the heterogeneity in the level of formality/casualness among clients, and that clients’ preference may depend on their own level formality.

Three studies surveyed psychiatric patients about their preferences on their medical practitioners, but they showed equivocal results. First, psychiatric patients perceived nurses in uniform as more of benevolent autocrat than nurses in street clothes (8). Second, Gledhill et al. (9) found that psychiatric patients preferred psychiatrists wearing formal attire [e.g., long-sleeved shirts with formal trousers and ties (male) and blouses and skirts (female)] with white coats, which were perceived as more competent and understanding. Third, conversely, most (96%) psychiatric patients in the study of Nihalani et al. (10) preferred psychiatrists not wearing white coats, and the majority of their participants preferred psychiatrists wearing casual shirts and pants/skirts.

Apart from the formality of therapists’ attire, few studies investigated the formality of the office setting of psychotherapy. Bloom et al. (11) asked 144 undergraduate students to sit at two settings of psychotherapy (traditional vs. humanistic) and rated therapists of different sexes based on their initial impression. Traditional office looked more formal, which included therapists sitting behind the desks (around 183 cm apart from the clients) with a five-drawer file cabinet next to the desks, and the walls of the office were decorated with four diplomas. In contrast, therapists sat face-to-face with the clients (around 89 cm apart from the clients) in humanistic office with a small end table between them. The walls were decorated with several posters with some poignant sayings. In general, humanistic office looked less formal than the traditional office. They found that participants perceived female therapists in the traditional office setting as significantly more credible than female therapists in the humanistic office setting. In contrast, participants perceived male therapists in the humanistic office as significantly more credible than male therapists in the traditional office. Later, Gass (12) asked 233 undergraduate students to listen to some audio recordings of psychotherapy while being presented with photos of therapists with formal or casual attire in two office settings: behind desk (more formal) vs. without desk (less formal). Based on participants’ initial impression, therapists with casual attire were perceived as more personally attractive when seated without desk. Findings of these two studies on the formality of office setting suggested that informality/casualness can sometimes be perceived positively as an initial impression; however, participants of both studies were only undergraduate students, so their results may not be representative of real clients of psychotherapy. Also, their results may only be indicative of initial impression, which may not be generalized to later stages of psychotherapy.

The above literature review has shown confounds in the preference for the formality of psychotherapy in terms of therapists’ attire and office setting. Halmagyi (5), a recent qualitative study that interviewed therapists, concluded that therapists’ attire is an area that is absent in formal mentioning in training and is believed to be a common assumption in the field; however, common agreement and awareness on attire are both lacking, not to mention the formality of psychotherapy. Therapists dressing formally (casually) and having a formal (casual) office setting do not imply therapists conducting psychotherapy formally (casually), especially since attires and office settings are fixed variables in psychotherapy, but there can be dynamical changes in the therapist-client relationship where the formality of psychotherapy changes throughout the course of psychotherapy. A preference on the formality of psychotherapy based on initial interaction may not be representative of that in later stages of psychotherapy. Moreover, formality exists over a spectrum/continuum, rather than dichotomously (5). Fixed variables such as attire and office setting may qualify the measurements of formality to few dichotomous/binary categories (e.g., formal vs. casual).

Furthermore, even though attire and office setting contribute to the perception of formality, a crucial component of formality is constituted by the way people speak (i.e., the use of language) (13). Language, as a tool and method of information delivery, does not only encode the message itself, but also the paralanguage and metacommunication of the speakers, such as speakers’ emotions, attitudes, intentions, and nuanced meanings. From a pragmatic point of view, a dialogue is a communication activity between parties that involves the interplay between speakers, rather than a mere summation of independent monologs. For example, the English fillers (e.g., *um*, *uh*, etc.) and discourse markers (e.g., *you know*, *I mean*, etc.) are analogs to the “traffic lights” in a conversation, signaling a deceleration of conversation, changing topic, or turn taking to the interlocutors (14–16). A recent study by Jin and Tay (17) pioneered in investigating the occurrence of Mandarin particles *ou* and turn-construction-units (TCU) in clinical contexts. They found that non-TCU-final *ou* occurred mainly in the patient speech to mark newsworthiness and call for the doctor’s attention, while TCU-final *ou* occurred in both the doctor and the patient speech serving various functions. This demonstrates how “subtle” use of particles can facilitate relationship building in conversational activities. In short, pragmatically speaking, language encode paralinguistic and metacommuncative information. As psychotherapy is primarily a speech-oriented activity, it is thus believed that linguistic/pragmatic analysis is crucial for capturing the dynamics of formality in psychotherapy. For instance, the changes in formality in the psychotherapeutic interaction can be reflected by the formality in the speech of therapists and clients.

In linguistics, *discourse particles* are argued to be the speech markers of formality. In contrast to grammatical particles (e.g., *up* and *down* in phrasal verbs and infinitival *to* in English), discourse particles (e.g., *well*, *now*, sentence-final *huh?*, and sentence-final *man!* in English) are defined as particles that express speakers’ attitudes, including their emotions, expectations, intentions, and assumptions, which are crucial to steer the flow and interaction of a dialogue (18, 19). Their meanings and pragmatic functions are so abstract and idiosyncratic that are often described as “untranslatable”; nevertheless, their frequency in ordinary speech is high. The communicative competence is considered drastically impaired if learners of language cannot master the use of particles (18, 20). The following examples demonstrate how discourse particles encode speakers’ attitudes in Cantonese, the language used in the psychotherapy sessions of the current study. Cantonese discourse particles are also known as sentence-final particles and utterance-final particles, for they typically appear at the end of sentences. The sentence-final particles in examples (1–3)^1^ convey different pragmatic meanings and discourse functions using the same sentence body *keoidei taisyu* (“they read books”): *me* in (1) transforms the statement into a rhetorical question; *gwaa* in (2) signals speculation; and *lo* in (3) expresses obviousness.

**Table:** 

(1)	*Keoidei taisyu* ***me**?*
	they read.book particle
	“Do they really read books?” [Rhetorical question]
	
	
(2)	*Keoidei taisyu* ***gwaa***.
	they read.book particle
	“I guess they read books.” [Speculation]
	
	
(3)	*Keoidei taisyu* ***lo***.
	they read.book particle
	“It is needless to say that they read books.” [Obviousness]

Since Cantonese particles typically occur at the end of sentences, they are argued to be employed for recalibrating and finalizing speaker’s epistemic stance, including reaffirming and modulating (upgrading or downgrading). For instance, *gwaa* in (2) is a particle indicating speaker’s uncertainty, and *lo* in (3) is a particle indicating speaker’s certainty. It is worth noting that language often has multiple strategies to encode similar discourse functions, and the methods can interact with each other. For instance, Chor (21) argued that Cantonese particles are used to recalibrate the epistemic stance of the speakers laid down by other strategies, including epistemic adverbials (e.g., *probably*, *certainly*, etc.), modals (e.g., *must*, *may*, etc.), and epistemic phrases (e.g., *I think*, *I believe*, etc.). However, pragmatically, nuances of formality are incidentally encoded by different strategies. For instance, both the adverbial “obviously” and the Cantonese particle *lo* encode obviousness. While adverbials are mostly emotionally neutral, the use of particles always brings in additional speaker’s emotions/attitudes and subjective mood, and the exudation of personal emotions is often considered more causal in terms of formality than staying emotionally neutral. In other words, to avoid personal emotions and mood, formal speech tends to reduce the use of particles and employ other more neutral strategies, say adverbials (19, 22–28).

Cross-linguistically, linguists are able to find corresponding “redundant” adverbials to particles in different languages that encode very similar semantic/pragmatic meanings. Such a correspondence system in syntax-discourse mapping is argued to be universally true in all languages (22, 25–29). It is also widely observed that particles in different languages generally encode semantic/pragmatic meanings with higher casualness, and higher usage of particles is cross-linguistically observed in casual/colloquial speech (e.g., conversation with close friends and relatives) (22, 30–35). Syntactically speaking, discourse particles do not belong to the argument structure in syntax. They are “optional elements” in sentences (vis-à-vis obligatory elements: subjects, verbs, and objects). Given such optionality, particles are reduced to the minimum to preserve a high degree of formality in formal speech (e.g., reporting speech) (22, 30–35). Thus, the usage of particles in speech has been an effective quantifier of the formality of the discourse: the more casual the discourse, the higher the usage of particles. For instance, based on naturalistic data of Cantonese, Leung (32) reported that particles took up 0–6% of utterances in reporting speech (e.g., news report, weather report, transportation report, etc.), 29–33% of utterances in radio/television commentary and interviews, and 62–71% of utterances in chit-chat and daily conversation. Based on the varying use of particles across Cantonese speeches that vary in formality and the cross-linguistic observation on the relationships between particles and formality, we hypothesize that the use of particles as a quantifier of the formality of a discourse, including the formality of psychotherapy sessions: the more casual the discourse, the higher the usage of particles.

Formal speech is generally characterized by the neutrality of speakers’ emotions/mood. In psychotherapy, neutrality as a therapeutic stance originated within psychoanalysis and was viewed as one of the most effective viable therapist stances for many decades (36). In essence, psychoanalysts’ neutrality helps to maintain the therapeutic boundary, and prevent the therapists’ values and feelings from interfering with clients’ exploration and the therapeutic process (37–39). However, psychoanalysts are also aware of the importance of empathy and engagement in a real relationship with patients, noting that the gratification of patients’ needs for affection and dependency (among other needs) should be provided at times to motivate them to continue to work on understanding themselves in deeper ways (36, 40–43). In other words, there needs to be a balance between neutrality of emotions and the exudation of emotions/empathy in classical psychoanalysis (36, 39, 42).

Later, it is similarly proposed in other therapeutic approaches, such as psychodynamic, humanistic, and integrative approaches, that therapeutic *neutrality* should be enacted within the context of *empathic atmosphere* to be effective (36, 44–47). The empathic atmosphere first involves the therapists to experience empathy (i.e., entering the patients’ inner worlds, intellectually grasping the worlds, and to an extent affectively experiencing them). At the same time, the therapists do not lose themselves in the clients’ worlds, but instead maintain their separate identities. Most importantly, the therapists are able to communicate their empathy via their verbal responses to facilitate patients’ inner experiencing and emotional expression in the here and now, without pulling clients to experience more affect than what they are ready for through excessive warmth (36, 40, 41).

In summary, therapists’ verbal response of empathy to clients involves both neutrality/refrainment and the exudation of personal affect/warmth. Whether the therapists are expressing neutrality vs. warmth can be reflected in the therapists’ speech, for examples, the pragmatic and paralinguistic/metacommunicative aspects of speech encodes speakers’ emotions and attitudes. These aspects may in turn reflect in the formality of the speaker. The current study aims to investigate the linguistic elements in speech that can capture, pragmatically, the formality in psychotherapy. As a first step, we explore how these linguistic elements reflecting the speakers’ formality relates to clients’ experience of therapist empathy.

Clients’ experience of therapist empathy has long been hypothesized to be a key process in psychotherapy contributing to client change (47, 48). It can be defined broadly as “the therapist’s sensitive ability and willingness to understand the client’s thoughts, feelings and struggles from the client’s point of view” (49). A recent meta-analysis on 82 independent samples and 6,138 clients has shown that empathy is a moderately strong predictor of therapy outcome (48). Linguistically, both intonation (the pitch pattern of a sentence) and particles perform similar pragmatic functions of encoding speakers’ emotions and attitudes (19, 22–24, 29, 34, 50–54) and are linguistic features that are pertinent to empathy. It is argued that all languages use a combination of intonation and particles for expressing connotative meaning, and they form a continuum in world languages: languages that use more intonation tend to use fewer particles, and vice versa. For example, languages that primarily have larger inventories of intonation, such as English and French (e.g., *hein?*), have fewer discourse particles. In contrast, languages that use a large number of particles, such as Cantonese, German (e.g., *doch*, *eben*, *ja*, etc.), Japanese (e.g., *ka*, *ne*, *yo*, etc.), Mandarin (e.g., *ba*, *le*, *ma*, etc.), and Vietnamese (e.*g., à, hả*, *nhỉ*, etc.), have smaller repertoires of intonation (19, 32, 55, 56). However, previous studies on psychotherapy only investigated intonation, but the role of particles is left unexplored. For example, the use of intonation by therapists was annotated in the transcript excerpts of psychotherapy sessions in studies of conversation analysis, but intonation was not the center of their discussion and the intonation mentioned was mostly certain one-off instances in speech (57–59). Weiste and Peräkylä (60) was the first study that investigated the systematic use of intonation by the therapists in psychotherapy. They concluded that therapists tend to continue the intonation of clients with a lower pitch and softer voice when they validate clients’ opinions and change to using a higher pitch and louder voice when they challenge clients’ views. Since then, more studies investigated the use of continuity of clients’ intonation by the therapists in psychotherapy (61–63).

Such a concept of continuity resembles the notion of synchrony in the studies of empathy and psychotherapy. First, empathy has been conceptualized as a general process that involves mirroring, which depends on a process of synchrony and imitation (64). Second, there has been a history of studying interpersonal synchrony, which is argued to promote cooperative behavior, affiliation, and compassion. The alignments can occur in levels of neural, perceptual, affective, physiological, and behavioral responses during social interaction (65–69). Specifically in psychotherapy, since therapists assist clients through verbal interactions, previous studies showed relationships between dyadic synchrony and the quality of interpersonal interactions in therapist-client dyads in psychotherapy. For example, higher synchrony between therapists’ and clients’ body movements was associated with higher ratings of relationship quality and treatment outcome (70). Higher synchrony in physiological signals, such as skin conductance, between dyads was associated with better therapist empathy (71, 72). In terms of language use, linguistic synchrony between the therapists and clients are hypothesized to be beneficial to the treatment outcome therapists and clients may attain synchrony by developing “common language” by mutual adaptation to each other’s linguistic behaviors (73). For instance, synchrony in language style between dyads was found to be associated with higher therapist empathy and better treatment outcome (74, 75). Also, Imel et al. (76) found that synchrony in vocally encoded arousal (measured by mean fundamental frequency) between dyad members was higher in sessions with high empathy ratings; however, Gaume et al. (77) failed to replicate their results. They attributed the failure of replication to three major reasons. First, Imel et al. investigated standardized patients (i.e., actors portraying generic patients) who may amplify a hypothesized synchrony effect. Second, the clinicians in the study of Imel et al. were recently exposed to training of Motivational Interviewing and might be particularly attentive to empathic reflection of patient emotions. Third, there are differences in language and culture. The sessions of Gaume et al. were held in French-speaking Switzerland, while those of Imel et al. were held in English-speaking United States. Paralinguistically, Americans tend to speak more loudly than Europeans, and English has more fluctuating prosody and pitch than French, so English may show synchrony in prosody more easily.

Unfortunately, existing approaches to linguistic synchrony tend to be methodologically divided—either quantitative or qualitative approach (78). Quantitatively, automated text analytic software like Linguistic Inquiry and Word Count (LIWC) (79) is widely employed to analyze the frequency count of content words and grammatical words in overall sessions, which claims to reflect analytical thinking, clout, authenticity, and emotional tone of the speakers. In general, mixed-effects modeling and cluster analysis are proposed to be the common statistical analytics (78–86). However, discourse particles are not considered in the calculation LIWC. Qualitatively, conversation analysis is a common method to investigate the turn-by-turn linguistic features between the therapists and clients (14, 15, 17, 60, 63, 78, 86–90). Tay and Qiu (78) and Qiu and Tay (86) thus proposed that mixed methods of both quantitative and qualitative approaches should be employed in the linguistic analysis of psychotherapy to capture both the generalizable patterns in higher levels (e.g., session level) and lower levels (e.g., turn level). We follow Qiu and Tay (86) in applying mixed-effects modeling in our subsequent analysis of discourse particles, followed by qualitative conversation analysis of examples in our data.

The current study explores how the use of particles, an indicator of the formality of speech in psychotherapy, is related to ratings of therapist empathy. Based on the findings of the previous studies, we can hypothesize at least three relationships between the use of particles, hence the formality of psychotherapy, and therapist empathy. First, from the perspective of synchrony of features, previous studies have shown that higher synchrony in linguistic features and other non-verbal features is associated with higher therapist empathy and better treatment outcome (64, 70–72, 74–76). This predicts that synchrony of particle usage (and hence formality) between clients and therapists is associated with higher therapist empathy.

Second, from the perspective of formality, most studies based on binary options of absolute formality suggested that clients prefer formality over casualness in psychotherapy settings and the therapists’ attire (1–3, 6). In addition, although particle use may have interactional component and be dependent on their interlocuters, particle use can also be a characteristic of each individual (91). If these findings also apply to the formality of psychotherapy speech, it predicts that, regardless of the formality or casualness of the clients, the lower the usage of particles by the therapists (i.e., therapists being more formal), the higher the therapist empathy.

Third, by contrast, relative formality (instead of absolute formality) of psychotherapy can be important to clients’ perception (7). If findings about relative formality prevails in psychotherapy speech, it predicts that fewer use of particles by the therapists than their clients, hence indicating higher therapist formality than their clients, predict higher therapist empathy. Also, clients can have different degree of formality/casualness (7), and some studies suggested that therapist casualness may play a role in the initial impressions (11, 12). Preference for therapist formality may thus differ between clients of different degree of formality and across different stages of psychotherapy.

## 2 Materials and methods

### 2.1 Setting

Data were collected from a department training clinic in a university in Hong Kong, where master’s level trainees complete their first 20-weeks counseling practicum as part of the degree requirement. They also participated in individual and group supervision as part of training. As such, the data represented the therapists’ work with one of their first few clients. They had one year of counseling training prior to starting the counseling practicum. Their training was based on Hill’s (92) three-stage model of helping. Based on this model, counselors learned to use basic helping skills from client-centered, psychodynamic, and cognitive-behavioral orientations in the counseling sessions.

The clinic provided low-fee psychotherapy for adults living in the community, and sessions were conducted weekly over the practicum period. The therapists and clients were mainly matched by availability, although sometimes the therapist can indicate their interest to work with specific clients based on the clients’ issues that they indicated at screening. Using the standard of Bloom et al. (11), the setting of the counseling rooms in the clinic was all identically humanistic: therapists sat face-to-face with clients with a small end table between them.

### 2.2 Participants

Thirty-nine (29 women, 10 men) clients were included in this study. Clients’ age ranged from 18 to 57 years (mean = 34.67, *SD* = 10.85). Thirty-nine (31 women, 8 men; mean age = 34.25, *SD* = 8.01) therapists provided counseling to the 39 clients, thus forming 39 unique therapist-client dyads. All therapists and clients were Asians, and the counseling sessions were conducted in Cantonese.

### 2.3 Measures

The Therapist Empathy Scale (TES) (93) is a nine-item observer-rated measure of therapist empathy. The items cover the cognitive, affective, attitudinal, and attunement aspects of empathy. Each item is rated on a seven-point scale from 1 = *not at all* to 7 = *extremely*, and a score is given to each item after observers complete watching a videotaped counseling session. A sample item is “Expressiveness: A therapist’s voice demonstrates expressiveness when the therapist speaks with energy and varies the pitch of his or her voice to accommodate the mood or disposition of the client.” The total score (range from 9 to 63) is used in this study, with a higher score indicating higher observer-rated therapist empathy. The TES has excellent psychometric properties in the scale development sample (93), and its internal consistency for the current sample is high (α = 0.96).

In the present study, eight raters were first trained on the TES using eight videotaped counseling sessions unrelated to the study sample but were collected from the same setting. Based on these sessions, the intraclass coefficient (ICC) for the eight raters was 0.79. Using Cicchetti (94)’s guidelines on interrater reliability, where ICCs < 0.40 are poor, 0.40–0.59 are fair, 0.60–0.74 are good, and > 0.75 are excellent, the raters are considered to have excellent interrater reliability. The raters then proceeded to rate counseling sessions from the study. As a reliability check, about 40% (61 sessions) of the videotapes were rated by two raters. The ICC based on a mean-rating (*k* = 2), consistency, two-way random effects model was 0.90, indicating excellent interrater reliability beyond the training phase. No outliers were detected in our sample as the scores all fall within ± 3.29 SD (95), which indicates that there were not very unempathic therapists in our data.

### 2.4 Procedures

Potential clients learned about the clinic through website listings, flyers, and word-of-mouth. The clients reported a range of issues that they sought counseling for during initial screening intake, including mood difficulties, stress, personal growth, family issues, interpersonal problems, academic/work-related problems, and marital/romantic relationship issues.

Following Flückiger et al. (96), four sessions (representing early, mid, and late stages of psychotherapy) were randomly selected from the 12 sessions of each dyad (i.e., a total of 156 session) for the research purpose of this study. Different tasks were involved in different stages of the therapy in this convention of dividing therapy stages. Typically, emphasis was given in the early stage to establish the working alliance between the therapists and the clients. The middle stage involved deeper work on the clients’ issues, and the late stage involved helping the clients look forward to life independent from the therapists and getting the clients ready to part from the therapists.

All analyzed sessions were videotaped and voice recorded. Trained observers completed the TES after watching the videotapes of the sessions.

In addition, the videotapes and voice recordings of all sessions were manually transcribed by university undergraduate students, who were native speakers of Cantonese. They adopted the same convention of transcription, including the delimitation of utterances and speaker turns based on native judgments. After that, all transcripts were cross-checked with voice recordings by native adult speakers of Cantonese who were trained in Cantonese phonetics. They focused on the accuracy of the transcription of particles with reference to the lists of Cantonese discourse particles compiled by Tang (22) and Sybesma and Li (52). The usage of particles by the clients and therapists in each session was calculated by the percentage of utterances produced with particles by the clients or therapists = (number of utterances produced with particles by the client or therapist in a specific session/total number of utterances produced by the client or therapist in the specific session) × 100%. The same delimitation of utterances and speaker turns was previously employed by Lee et al. (97) in their linguistic analysis of discourse boundaries.

Based on the obtained particle usage data, we first calculated the synchrony of the use of particles by obtaining the absolute value (i.e., ignoring directions of subtraction) of the pairwise subtraction between clients’ and therapists’ percentage usage of particles in each session. The closer the value is to zero, the higher the synchrony of the use of particles by the dyad, hence having higher synchrony in formality. Second, we calculated the relative usage of particles by the therapists by pairwise subtraction of clients’ percentage usage of particles from therapists’ percentage usage of particles in each session. The higher (more positive) the relative usage by the therapists is, the more particles the therapists use than the clients, hence having higher casualness than their clients.

### 2.5 Multilevel modeling

Descriptive and bivariate statistical analyses were conducted using R (98). In addition, session-level data were nested within the client-level data, but there was no further nesting within therapist-level data since the therapist-client dyads are all unique. Multilevel modeling was performed on our data using *lme4* package (99) in R with TES as the dependent variables of three multilevel models. Model 1 investigated the synchronized use of particles, which included predictors of the synchrony of the use of particles between the therapists and clients and session number. Model 2 investigated the usage of particles by the therapists regardless clients’ usage (i.e., absolute formality in psychotherapy), which included predictors of the usage of particles by the therapists and session number. Model 3 investigated the relative use of particles (i.e., relative formality in psychotherapy), which included predictors of usage of particles by the clients, relative usage of particles by the therapists, and session number. We also included random slopes of clients [equivalent to unique therapist-client dyads (100–103)] for the (absolute/relative) usage of particles in each model. As measures of effect size, both the proportion reduction in error resulting from adding predictors (104) and *f*^2^ adjusted for multilevel models (105) were computed. The *f*^2^ for multilevel models can be interpreted as follows: ≥ 0.02 are small, ≥ 0.15 are medium, and ≥ 0.35 are large (106).

### 2.6 Qualitative conversation analysis

Following the convention of mixed methods study in psychotherapy (78, 86), we analyzed two qualitative examples in our data to elaborate specific examples at turn level based on our quantitative findings at a more “global” session level.

## 3 Results

### 3.1 Descriptive statistics and intercorrelations

Table 1 reports the session-level means, standard deviation, range, and the bivariate correlations among the synchrony of the use of particles, usage of particles by the clients, usage of particles by the therapists, relative usage of particles by the therapists, and TES. Although the usage of particles by the therapists is correlated positively with that by the clients (*r* = 0.44) and negatively with TES (*r* = −0.18), there is no correlation between synchrony of the use of particles and TES. In contrast, relative usage of particles by the therapists correlates with both TES (*r* = −0.02) and clients’ usage of particles (*r* = −0.46), which allows for further interaction analyses.

**TABLE 1:** Session-level mean, standard deviation, range, and intercorrelations of study variables.

Variable	*N*	Mean	*SD*	Min.	Max.	1	2	3	4	5
1. Synchrony of the use of particles (|therapists’ % usage of particles—clients’ % usage of particles|)	156	7.01	5.00	0.02	20.89	−				
2. Usage of particles by the therapists (% of particles/total utterances produced)	156	26.27	8.47	2.29	49.78	0.08	−			
3. Usage of particles by the clients (% of particles/total utterances produced)	156	27.00	7.72	1.67	50.00	0.15	0.44***	−		
4. Relative usage of particles by the therapists (therapists’ % usage of particles—clients’ % usage of particles)	156	–0.73	8.59	–18.81	20.89	–0.06	0.59***	−0.46***	−	
5. Therapist Empathy Scale (TES)	156	38.80	7.89	18.00	56.50	–0.01	−0.18*	0.03	−0.02*	−

*p < 0.05, ***p < 0.001.

### 3.2 Multilevel models

#### 3.2.1 Model 1

Results of Model 1 show that there are neither significant main effects of the synchrony of the use of particles [*B* = 22.36, *SE* = 25.90, *t*(114.34) = 0.86, *p* = 0.39] and session number [*B* = 1.23, *SE* = 0.69, *t*(107.05) = 1.77, *p* = 0.08], nor significant interaction effects between the two predictors [*B* = −7.27, *SE* = 8.89, *t*(105.50) = −0.82, *p* = 0.42], on TES. This indicates that therapist empathy is not predicted by the synchrony in particle usage between clients and therapists, and hence the synchrony in formality, and time in therapy. As measures of effect size, the proportion reduction in error resulting from adding predictors in Model 1 is 2.30%, and the *f*^2^ is 0.04, indicating a small effect size of the predictors.

#### 3.2.2 Model 2

Similarly, results of Model 2 show that there are neither significant main effects of the usage of particles by the therapists [*B* = −0.38, *SE* = 13.65, *t*(24.45) = −0.03, *p* = 0.98] and session number [*B* = 2.34, *SE* = 1.28, *t*(70.75) = 1.83, *p* = 0.07], nor significant interaction effects between the two predictors [*B* = −6.08, *SE* = 4.68, *t*(71.47) = −1.30, *p* = 0.20], on TES. This indicates that therapist empathy is not predicted by the usage of particles by the therapists, hence the absolute formality in psychotherapy, and time in therapy. As measures of effect size, the proportion reduction in error resulting from adding predictors in Model 2 is 3.25%, and the *f*^2^ is 0.04, indicating a small effect size of the predictors.

#### 3.2.3 Model 3

**FIGURE 1:**
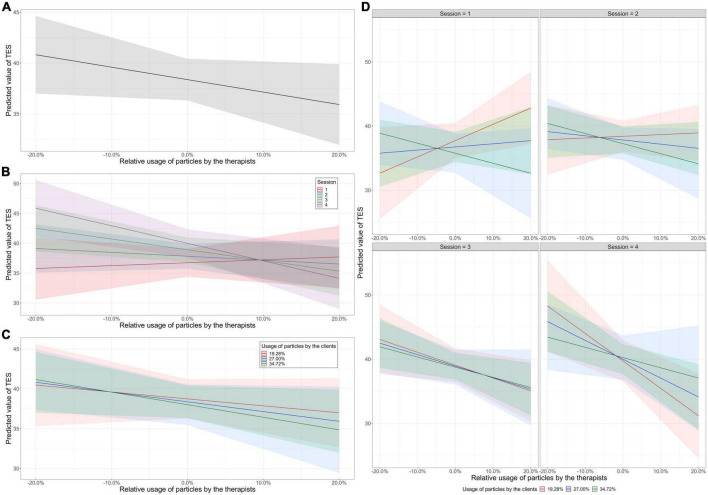
Results of multilevel modeling (Model 3). **(A)** Main effects of relative usage of particles by the therapists on TES scores. **(B)** Interaction effects between the relative usage of particles by the therapists and session number on TES scores. **(C)** Interaction effects between the relative usage of particles by the therapists and the usage of particles by the clients on TES scores. **(D)** Interaction effects between relative usage of particles by the therapists, the usage of particles by the clients, and session number on TES scores.

By contrast, results of Model 3 show that there are significant main effects of the relative usage of particles by therapists, *B* = 123.54, *SE* = 44.06, *t*(118.97) = 2.80, *p* = 0.006, on TES. Using the R package *sjmisc* (107), Figure 1A shows that the lower (more negative) the relative usage of particles by the therapists compared to their clients, the higher the predicted TES. This indicates that therapists who are more formal than their clients are perceived to have higher therapist empathy.

Second, there are significant interaction effects between the relative usage of particles by the therapists and session number on TES, *B* = −49.53, *SE* = 17.38, *t*(116.91) = −2.85, *p* = 0.005. Specifically, in early sessions (e.g., session 1; red line in Figure 1B), the higher the relative usage of particles by the therapists (i.e., therapists being more casual than the clients), the higher the predicted TES. On the contrary, in the middle and later sessions (e.g., sessions 2, 3, and 4; blue, green, and purple lines in Figure 1B), the lower the relative usage of particles by the therapists (i.e., therapists being more formal than the clients), the higher the predicted TES.

Third, there are significant interaction effects between the relative usage of particles by the therapists and the usage of particles by the clients on TES, *B* = −397.22, *SE* = 145.93, *t*(118.36) = −2.72, *p* = 0.007. Figure 1C shows an overall negative relationship between relative usage of particles and the predicted TES, regardless of the usage of particles by the clients, for all curves have negative slopes. The lower the relative usage of particles by the therapists (i.e., therapists being more formal than the clients), the higher the predicted TES. In addition, the slope becomes steeper when clients used more particles [red line: client particle usage at 1 *SD* below the mean (19.27%); blue line: client particle usage at the mean level (27.00%); green line: client particle usage at 1 *SD* above the mean (34.72%)]. In other words, the same unit of increment in the relative usage of particles by the therapists results in a greater decrease in TES scores as clients’ usage of particles increases (i.e., when clients are more casual).

Fourth, there are significant interaction effects between relative usage of particles by the therapists, the usage of particles by the clients, and session number, *B* = 141.17, *SE* = 60.00, *t*(117.48) = 2.35, *p* = 0.02, on TES. Figure 1D shows the results of the 3-way interactions.

For clients having high usage of particles (green lines, usage 1 *SD* above mean = 34.72%), the lower the relative usage of particles by the therapists, the higher the predicted TES across all sessions. In contrast, for clients having low usage of particles (red lines, usage 1 *SD* below mean = 19.28%), therapists’ lower relative usage of particles is associated with higher predicted TES only in the later sessions (sessions 3 and 4); therapists’ higher relative usage of particles is associated with higher predicted TES in the earlier sessions (sessions 1 and 2). In general, the slopes of clients having high usage of particles (green lines, usage 1 *SD* above mean = 34.72%) are consistently negative across sessions, whereas the slopes of clients having low usage (red lines, usage 1 *SD* below mean = 19.28%) and average usage (blue lines, mean usage = 27.00%) of particles become more negative as therapy proceeds. Specifically, the slopes of clients having low usage (red lines) change from positive in earlier sessions (sessions 1 and 2) to negative in later sessions (sessions 3 and 4). The slope of clients having average usage (blue lines) is initially positive in early session (session 1) but turns negative in later sessions (sessions 2–4). The interaction analyses suggest that if clients are casual, therapists are predicted to have higher empathy ratings if they speak more formally in all stages of psychotherapy. In contrast, if clients are formal (i.e., use fewer particles), therapists are predicted to have higher empathy ratings if they speak more *casually* than the clients in the early stage of psychotherapy; however, in the later stages of psychotherapy, therapists are predicted to have higher empathy ratings if they speak more formally than the clients.

There are no significant main effects of the clients’ usage of particles [*B* = −17.50, *SE* = 14.88, *t*(102.76) = −1.18, *p* = 0.24] and session number [*B* = −0.32, *SE* = 1.63, *t*(113.95) = −0.20, *p* = 0.85] on TES. The interaction effects between the usage of particles by the clients and session number on TES are also found non-significant, *B* = 5.19, *SE* = 6.13, *t*(115.25) = 0.85, *p* = 0.40.

As measures of effect size, the proportion reduction in error resulting from adding predictors in Model 3 is 9.32%, and the *f*^2^ of the model is 0.16, indicating a medium effect size of the predictors.

### 3.3 Qualitative conversation analysis

#### 3.3.1 Excerpt 1

Excerpt 1 is taken from a psychotherapy session at the mid stage, which discussed financial issues with client’s family members. Both the therapist (age: 38 years) and the client (age: 23 years) are male. The observer-rated TES of this session is 23 (possible TES ranges from 9 to 63; mean = 38.80, *SD* = 7.89). The session was conducted in Cantonese. We translated the transcript into English, while retaining Cantonese particles in Jyutping romanization (108) in bold font at the place where they were used with a bracket indicating the functions of the particles according to description in Tang (22) and Sybesma and Li (52).

1. Therapist: After listening to you, you have actually not received any money from them ***gaa wo*** [surprise].
2. Client: No ***aa*** [emotion softener], absolutely, except that I may have got several red pockets from them during the Chinese New Year.
3. Therapist: Yes ***lo*** [obviousness]. So, there is nothing about being realistic or not, isn’t it ***aa*** [emotion softener]?

Both the therapist and the client talked similarly casually with almost all utterances carrying particles. In fact, the sentences are still well-formed and grammatical even if all particles are removed. The addition of particles characterizes the mood of the speakers and adjust their emotions. For example, the use of *gaa wo* by the therapist in the first turn shows the therapist’s emotion of being surprised by new information that differs from his original epistemic stance. The *lo* the therapist used in the third turn expressed obviousness with a negative mood of looking down on the situation. Pragmatically, the whole sentence “Yes *lo*” can be translated as “it is needless to say that it is what I have predicted.” If we remove *gaa wo* and *lo* from the two sentences, the two sentences will become more emotionally neutral (i.e., without therapist’s subjective emotion and mood). The sentence in the first turn would then become a neutral description of the financial fact, and the “yes” phrase in third turn would be a genuine agreement.

#### 3.3.2 Excerpt 2

Excerpt 2 is taken from a psychotherapy session at the early stage. The therapist (age: 58 years) was male, and the client (age: 45 years) was female. The observer-rated TES of this session is 21. This therapist was observed with abundant use of compound particles, such as *aamaa*, which marked high casualness and speaker’s subjectivity and should be generally avoided in psychotherapy. In this session, the therapists produced 35 tokens of *aamaa*, while other therapists in our data produced an average of 0.67 token of *aamaa* in one session.

1. Client: So, he is arranged to sit with a student with dyslexia. That student… um… is not an able student. The teacher is kind to ask my son to teach and help the student.
2. Therapist: Your son is smart ***aamaa*** [obviousness]. That’s why the teacher asks him to help the student with dyslexia.
3. Client: But my son is not patient.

In turn 1, the client brought up the concern that his son was arranged to sit with a student with dyslexia. The therapist was unempathic to detect the anxiety of the client and assumed the client would be happy about her son being arranged to help with the neighboring student. The therapist used the particle *aamaa* in turn 2, which is carries even stronger subjective mood than *lo* in excerpt 1. In this case, it means that the therapist is certain that the client should have known that her son is smart, but the client is not aware of this, which carries a sense of irony and casualness (19, 22, 54). However, the client was actually unhappy about the seating arrangement at all, because she thought that her son was not patient enough to help other students (turn 3).

The overuse of the compound particles reflects high casualness in the therapist, and it is not an appropriate occasion to talk casually when the client was anxious about various aspects of her son in the early stage of psychotherapy (i.e., the therapist and client were not familiar with each other). The casualness in speech can be perceived as being unempathic. A component of empathy is communicative attunement, defined as “an active effort to stay attuned on a moment-to-moment basis with the client’s communications and unfolding experience” (48). Part of this attunement process involves being responsive to the clients’ preference for formality or casualness in the interaction. In other words, the client’s experience of therapist empathy probably includes how well their therapist responds to their preferred therapist demeanor (i.e., more or less formal). In the above scenario of a formal discussion about client’s son in an early stage of psychotherapy, talking casually can be perceived as disrespectful and playful, which may affect the perception of empathy. The therapist subjectively assumed the client was happy about the seating arrangement; however, the clients raised disagreement, which is believed that the use of compound particles in the excerpt helped little the client to reflect about her belief. The examples illustrate how the use of particles between turns, hence the formality of speech, may affect the perception of empathy.

## 4 Discussion

This study is the first study to explore the formality in the speech of psychotherapy by investigating therapists’ and clients’ use of discourse particles in relation to therapist empathy. Previous studies on psychotherapy mainly explored the formality of therapists’ attire and office setting using stimulated scenarios, which are fixed variables that may not capture the changes in formality throughout the course of actual psychotherapy. This study has explored a novel linguistic feature, the use of particles, which is the speech markers of the formality of discourse and can quantify the spectrum/continuum of formality.

First, in contrast to the previous findings on the association between synchrony of linguistic or non-verbal features and therapist empathy (64, 70–72, 74–76), our results (Model 1) suggested that the synchrony in the use of particles, hence the synchrony in formality, between clients and therapists is not associated with therapist empathy. These findings are in line with Gaume et al. (77) who failed to replicate the results of Imel et al. (76), which found that synchrony in vocally encoded arousal between dyad members was higher in sessions with high empathy ratings. Similar to Gaume et al.’s study, we used real clients rather than standardized clients, who were used in Imel et al. Real clients’ synchrony effects in linguistic features may not be as conspicuous as those in standardized patients (51). In addition, there can be cultural and paralinguistic differences in the use of particles across different languages that may have contributed to differences in findings across studies that deserve further her inquiry in future research. Second, our results (Models 2 and 3) cast doubt on clients’ preference for absolute formality of therapists (i.e., therapists being formal regardless of the formality or casualness of their clients). Results suggested that therapist empathy is generally observed when therapists are *relatively* more formal than the clients (i.e., lower relative usage of particles by the therapists), which echo the findings of Hubble and Gelso (7) based on therapists’ attire. The fact that casualness is generally not preferred can be attributed to the fact that formality of therapists is often perceived as credibility and expertness (1–3, 5–7).

Third, while previous studies on the formality of psychotherapy were based on initial interaction or first impression, which may not be representative of later stages of psychotherapy, our study has explored the dynamics of formality across different stages of psychotherapy and among clients of different degree of formality/casualness. In general, the lower the relative usage of particles by the therapists (i.e., therapists being more formal than their clients), the higher the predicted TES, and this negative association gets stronger over the course of therapy (Figure 1B). However, as Figure 1D shows, when clients used few particles and thus more formal, *higher* relative usage of particles by the therapists was associated with *higher* therapist empathy in the early stages of psychotherapy. Taken together, therapist casualness may be interpreted as therapist empathy as therapists help clients who speak formally with few particles ease into the therapeutic relationship. As therapy progresses, the expectation of therapist formality relative to the client may have led observers to rate therapist as more empathic when they speak with fewer particles than their clients, regardless of clients’ level of formality/casualness. Our results suggest that the examination of therapists’ and clients’ use of particles across different stages of treatment may illuminate dynamic interactional styles that facilitate or hinder the psychotherapy process. Our methods of using linguistic features such as particles to quantify the spectrum/continuum of formality advances previous research that examined formality in a dichotomously way (e.g., formal vs. casual).

Linguistically, apart from being the speech marker of formality of discourse, particles also perform pragmatic functions of encoding speakers’ emotions and attitudes similar to intonation (19, 22–24, 29, 34, 50–54). Previous studies only explored the use of intonation in psychotherapy (57–63). This study is the first study that explores the relationships between particles and empathy in psychotherapy. Compared to intonation, particles are more stable and reliable substitutes in linguistic analysis. The lack of invariance problem, which refers to the absence of reliable connections between phonemes (mental representations of sounds) and their highly varying acoustic signals attested in actual speech (109, 110), has been a hard problem in speech perception; of which, intonation is particularly notorious because its acoustic measures are often unreliable and incomparable between utterances (24, 111). There are also copious factors that influence speech sounds, such as physiological state, level of vocal effort, process of production, and individuals’ characteristics (112). The analysis of intonation cannot only rely on acoustic measurements but on the subjective judgments of native speakers which are highly labor-intensive, so many previous studies on psychotherapy could only remark one-off instances of intonation used by the therapists (57–59).

Unlike intonation, particles are words with fixed combinations of consonants and vowels (i.e., syllables) that can be unequivocally identified, quantified, and analyzed based on transcriptions. Thus, particles may serve as an alternative feature to intonation for linguistic analysis of human emotions and attitudes. A next step would be to investigate how the formality in relation to therapist empathy changes within a session as a session also comprises many stages (e.g., warming up, supportive therapy, intervention, conclusions, etc.). While we recognize how the quantitative aspect of the usage of particles can be indicative of the formality in speech, which is not easily represented by other linguistic elements, there is also a qualitative aspect of the usage of particles (i.e., different types/categories of particles). Each particle encodes specific semantic and pragmatic functions, which are representative of the mood, emotions, attitudes, intonation, expectations, and assumptions of the speakers. It is worthy for future examination on how the different types of particles (e.g., interrogative particles, imperative particles, declarative particles, particles with other pragmatic functions, etc.) being used and/or synchronized in different conversation topics, backgrounds, and cultures of psychotherapy. Furthermore, particles are words in speech that can be recognized automatically by machines and analyzed computationally, which can be a favorable feature for cutting-edge big data analysis in psychotherapy/psychology.

Clinically, this study has implications for therapists’ use of language and formality that can be applied to practice. As a matter of choice of words, therapists can attend to their use of particles in relation to clients’ usage and adjust the use of particles in psychotherapy by training efforts. This study also raises the awareness of the correlates of therapists’ formality across different stages of psychotherapy, which is not often mentioned in therapist training.

There are certain complications in the formality of the psychotherapy setting of the current study. We collected data from a department training clinic in a university in Hong Kong, where master’s level trainees complete their counseling practicum as part of the degree requirement. First, even though Hong Kong has been has historically received influence from the West that the overall sociocultural relationships may be more egalitarian than typical Asian cultures, studies found that traditional Chinese values and hierarchical collectivism are still deep rooted in the mentality of Hong Kong clients (113, 114). Although therapists are generally positioned as more superior than their clients in Asian psychotherapy setting (113–115), trainee therapists are not experts, which may affect the formality/casualness of the psychotherapy as a power differential rooted in Asian hierarchical culture. In the present study, the clients knew that the therapists were trainees, which might lower the perception of the therapists’ authority; however, the clinic was a low-fee clinic serving clients from less advantaged socioeconomic background, which might elevate the “status” of the therapists, especially when the clinic was housed in a prestigious local university. Furthermore, the sex and age of the therapists and the clients could also matter, but we had relatively few male therapists and clients in the sample and an almost equivalent mean age between therapists and clients for specific sub-group analyses. Further studies can explore how age, sex, professional status of the therapists and clients may affect the formality of psychotherapy.

The therapists in this study were all trainees in their first counseling practicum, and all psychotherapy sessions were conducted in Cantonese, which may limit the generalizability of this study. Despite this, future studies can explore the differences in formality between trainee therapists and experienced therapists as trainee therapists tend to be more formal, or sometimes overly formal, than experienced therapists. Since the empathy ratings in the study were given by observers in the session level, which may not be ideal to reflect moment-by-moment change in the perception of empathy. Based on the current findings, future works can ideally work on clients’ statement-by-statement basis, so that the direct use of formality is examined. As such, the formality/casualness in critical events can also be independently investigated, which may be another important topic in the study of formality in psychotherapy.

The sample size of therapist-client dyads of the current study is small, which limits our ability to look at therapist-level predictors. The sample size in the level of the series of sessions is also small, which may have weaker statistical inference than that drawn from all sessions. However, we believe that our study serves as a much needed preliminary investigation into the formality of psychotherapeutic speech and the roles of particles in psychotherapy. Besides, since particles exist in most, if not all, languages, this study can be replicated in psychotherapy settings of other languages and cultural backgrounds in future investigation. However, there is a potential limitation that measurement of formality based on linguistic features can be language dependent. In addition, formality and its expression are embedded in cultural contexts. Other sociocultural relationships factors, such as age, gender, professional status, etc., can affect the meaning of formality. Formality out of discourse or sociocultural context can mean not formality, but creating interpersonal boundaries, such as coldness in personality or aloofness in response.

## Data availability statement

The raw data supporting the conclusions of this article will be made available by the authors, without undue reservation.

## Author contributions

JL, HC, TL, and SL contributed to the conception and design of the study. JL performed the statistical analysis and wrote the first draft of the manuscript. HC wrote sections of the manuscript. DT and NL organized the database and extracted transcription data. All authors contributed to the manuscript revision, read, and approved the submitted version.

## Footnotes

1 First line of each example is the *Jyutping* romanization of Cantonese characters (108). Second line is the gloss. Third line is the English translation of the whole sentence.
